# Vaginal Immunization to Elicit Primary T-Cell Activation and Dissemination

**DOI:** 10.1371/journal.pone.0080545

**Published:** 2013-12-05

**Authors:** Elena Pettini, Gennaro Prota, Annalisa Ciabattini, Alessandro Boianelli, Fabio Fiorino, Gianni Pozzi, Antonio Vicino, Donata Medaglini

**Affiliations:** 1 Laboratorio di Microbiologia Molecolare e Biotecnologia (LA.M.M.B.), Dipartimento di Biotecnologie Mediche, Università di Siena, Siena, Italy; 2 Dipartimento di Ingegneria dell'Informazione (DII), Centro per lo Studio dei Sistemi Complessi (CSC), Università di Siena, Siena, Italy; Commissariat a l'Energie Atomique(cea), France

## Abstract

Primary T-cell activation at mucosal sites is of utmost importance for the development of vaccination strategies. T-cell priming after vaginal immunization, with ovalbumin and CpG oligodeoxynucleotide adjuvant as model vaccine formulation, was studied *in vivo* in hormone-synchronized mice and compared to the one induced by the nasal route. Twenty-four hours after both vaginal or nasal immunization, antigen-loaded dendritic cells were detected within the respective draining lymph nodes. Vaginal immunization elicited a strong recruitment of antigen-specific CD4^+^ T cells into draining lymph nodes that was more rapid than the one observed following nasal immunization. T-cell clonal expansion was first detected in iliac lymph nodes, draining the genital tract, and proliferated T cells disseminated towards distal lymph nodes and spleen similarly to what observed following nasal immunization. T cells were indeed activated by the antigen encounter and acquired homing molecules essential to disseminate towards distal lymphoid organs as confirmed by the modulation of CD45RB, CD69, CD44 and CD62L marker expression. A multi-type Galton Watson branching process, previously used for *in vitro* analysis of T-cell proliferation, was applied to model *in vivo* CFSE proliferation data in draining lymph nodes 57 hours following immunization, in order to calculate the probabilistic decision of a cell to enter in division, rest in quiescence or migrate/die. The modelling analysis indicated that the probability of a cell to proliferate was higher following vaginal than nasal immunization. All together these data show that vaginal immunization, despite the absence of an organized mucosal associated inductive site in the genital tract, is very efficient in priming antigen-specific CD4^+^ T cells and inducing their dissemination from draining lymph nodes towards distal lymphoid organs.

## Introduction

T-cell priming at mucosal sites is of primary importance for the development of mucosal vaccine formulations and prime-boost strategies aimed to elicit mucosal and systemic effector immune responses. Despite the many attractive features of mucosal vaccination, only few vaccines approved for human use are currently administered mucosally, and they are all based on live attenuated microorganisms with none on subunit of pathogens [1]–[4]. To advance the development of new mucosal vaccines, not only appropriate antigens, adjuvants and delivery systems but also proper mucosal routes of administration should be deeply characterised [1], [5]–[7].

The induction of mucosal immune responses requires the presence of a mucosa-associated lymphoid tissue that provides a continuous source of B and T cells to mucosal effector sites [8]. Inductive sites for mucosal immunity consist of organized mucosa-associated lymphoid tissue as well as local and regional draining lymph nodes (LNs), whereas the effector sites include distinctly different histological compartments, mainly consisting of the lamina propria of various mucosae [9]. Inductive sites in the gastro-intestinal and respiratory tracts have been well defined and are composed by aggregated lymphoid tissues (gut-, nasal- and bronchial-associated lymphoid tissues respectively) and mucosa-associated lymph nodes (mesenteric and mediastinal lymph nodes), on the contrary the vaginal mucosa is devoid of histologically demonstrable organised mucosa-associated lymphoid tissue and the role of inductive site is played directly by draining iliac lymph nodes [5].

Female genital tract has therefore some unique features that should be taken in consideration in the development of vaccination strategies. Following vaginal immunization T-cell priming occurs in the iliac lymph nodes from which T and B cells migrate to the effector sites [5], [6], [10]–[12]. Antigens are sampled from the vaginal lumen by macrophages and dendritic cells (DCs) that then move towards draining iliac lymph nodes. Antigen-uptake across the vaginal mucosa barrier and immune responses in the genital tract are greatly regulated and influenced by the hormonal state and estrus phase [13]–[16]. Sex hormones also affect the migration of macrophages and DCs as well as T and B cells by involving the expression of adhesion molecules and chemotactic factors [17]. In particular, there are indications that estradiol, which induces an estrus-like state, inhibits T-cell priming preventing antigen loading by vaginal antigen presenting cells (APCs) after vaginal immunization, while progesterone, inducing a diestrous phase, facilitates antigen-uptake and T-cell activation [18], [19]. Hormonally mediated variations through the estrous cycle act also on the mucosal cell number and subset, having important implications in the antigen-uptake and subsequent T-cell priming [14], [19]–[22]. The phase of the menstrual cycle is indeed carefully taken into consideration in clinical trials of vaginal immunization (source www.clinicaltrials.gov).

The nasal route of vaccination targets the lymphoid tissue associated with the nasopharynx (NALT) and is recognised as an efficient inductive site capable of disseminating primed cells also to distal sites [10], and stimulate an immune response also in distal effector sites and systemically [8], [10], [23]–[28]. Previous studies conducted in this laboratory have deeply characterised the antigen-specific T-cell priming following nasal immunisation [29]–[32].

To overcome the limitation of the very low number of antigen-specific T cells *in vivo* [33], we have employed the adoptive transfer system of antigen-specific transgenic T cells into immunocompetent mice [34], [35]. By using this method, we have characterized T-cell clonal expansion and dissemination following nasal immunization showing that divided T cells, generated in the nasal associated lymphoid tissues and in draining cervical and mediastinal lymph nodes, migrate towards distal lymphoid organs, such as iliac and mesenteric lymph nodes and the spleen [31], [35]. The entry of nasally primed T cells into iliac lymph nodes was also shown to be strictly CD62L-dependent, while homing to mesenteric lymph nodes is regulated by both CD62L and α4β7 molecules [32].

For studying *in vivo* T-cell priming events, mathematical models represent an attractive tool to obtain quantitative information on the rates of division, death and migration. The application of mathematical models can indeed be highly relevant to analyse antigen-specific T-cell primary clonal expansion, based on the dilution of the carboxyfluorescein diacetate succinimidyl ester (CFSE) dye. It is well established that natural T-cell proliferation is inherently a stochastic phenomenon [36]. In stochastic models, cells are considered to act independently and divide or die accordingly to probabilistic rules. The Multi-type Galton Watson branching process is a prototypical branching process representing the development of a population whose members reproduce and die subject to random laws [37]. Quantitative analysis of CFSE data through mathematical models has been previously employed for studying *in vitro*, but not *in vivo*, cell proliferation [38]–[40]. Indeed, *in vivo* analysis raises several difficulties, mainly due to the fact that a lymph node is not an ‘isolated’ site but is part of the complex immunological system.

In the present work, we investigated the antigen-specific CD4^+^ T-cell primary activation following vaginal or nasal immunization with the model vaccine formulation constituted by ovalbumin (OVA) plus CpG oligodeoxynuclotide (ODN) 1826 as adjuvant. CpG is a Toll like receptor 9 ligand, that has been extensively described as an effective adjuvant for mucosal immunisation [41]. The distribution of OVA-bearing DCs at early time points following immunization was analysed using a fluorescent antigen. Primary activation and dissemination of antigen-specific T cells was studied *in vivo* after adoptive transfer of OVA-specific transgenic CD4^+^ T cells. Clonal expansion of transgenic T cells was evaluated in draining and distal lymph nodes and in the spleen at different time points. We adopted a multi-type branching process with immigration to model T-cell proliferation *in vivo* and estimated the probability of a cell to enter in division, rest in quiescence or migrate/die following both vaginal and nasal immunization.

## Materials and Methods

### Mice

Nine week old female OT-II [42] TCR-transgenic mice (H-2^b^) and C57BL/6J mice were purchased from Charles River (Lecco, Italy). Animals were maintained under specific pathogen-free conditions in the animal facilities at the University of Siena, and treated according to national guidelines (Decreto Legislativo January 27, 1992 n. 116, implementing 86/609/CEE Directive). All animal studies were approved by the Ethics Committee “Comitato Etico Locale dell'Azienda Ospedaliera Universitaria Senese” and the Italian Ministry of Health (number 4/2011, July 20, 2011).

### Antigen uptake by DCs

Six days before immunisation, the estrous cycle of mice was synchronized in the diestrous phase by subcutaneous injection of medroxyprogesterone (Depo-Provera, Pfizer, Italy) (3 mg/mouse) or in the estrous phase by administration of ß-estradiol valerate (Sigma–Aldrich) (0.1 mg/mouse). The stage of estrus was confirmed by vaginal smear. OVA uptake was tracked *in vivo* using ovalbumin-Alexa fluor 647 conjugate (Invitrogen Molecular Probes, Eugene, OR, USA). C57BL/6J mice were lightly anaesthetized by intraperitoneal injection of tiletamine, zolazepam hydrochloride (Zoletil 20, Laboratoires Virbac, France, 6 mg/kg) and xylazine (Xilor 2%, Bio 98 Srl, Italy, 3 mg/kg), and immunised by vaginal or nasal route with the fluorescent OVA (25 µg/mouse) mixed with the mucosal adjuvant CpG ODN 1826 (TCC ATG ACG TTC CTG ACG TT, Eurofins MWG Operon, Ebersberg, Germany) (20 µg/mouse). Mice immunized by vaginal route were housed in single cages and equipped with a collar for some hours to avoid self sniffing.

Groups of four animals were sacrificed 12, 24 and 72 hours after mucosal immunization. Iliac (subiliac, medial and external), cervical (superficial and posterior) lymph nodes and spleen were harvested and digested in Click's medium (Sigma-Aldrich) with 2 mg/ml of Collagenase D (Roche Applied Science, Penzberg, Germany) for 30 min at 37°C. Organs were mashed onto nylon screens (Sefar Italia, Italy) and washed in PBS with 0.5% of bovine serum albumin (BSA, Sigma-Aldrich) and 2 mM EDTA (Mallinckrodt Baker, Philipsburg, NJ, USA).

### Adoptive transfer of transgenic CD4^+^ T cells

Single cell suspensions from the spleen and pooled lymph nodes (cervical, brachial, axillary, mesenteric and iliac) of OT-II transgenic mice were enriched for CD4^+^ T cells, by negative selection using the EasySep magnetic nanoparticles (StemCell Technologies, Vancouver, BC, Canada), according to the manufacturer's protocol. The purity of the CD4^+^ T-cell population in the enriched fraction was >95%, as determined by flow cytometric analysis. OT-II transgenic CD4^+^ T cells were stained with CFSE (7.5 µM, Invitrogen) [43], for 10 min at 37°C. An amount of 2.5×10^6^ of CFSE-labelled T cells were injected into the tail vein of each recipient mouse.

### Vaginal and nasal immunization of mice and sample collection

Twenty-four hours after adoptive transfer of CFSE-labelled OT-II CD4^+^ T cells, progesterone treated C57BL/6J mice were vaginally or nasally immunized with OVA grade V (Sigma-Aldrich) (25 µg/mouse) and CpG ODN 1826 (20 µg/mouse). Mice were lightly anaesthetized by intraperitoneal injection and then inoculated with OVA and CpG into the vagina or into nostrils with a volume of 15 µl. Mice immunized by vaginal route were housed individually and equipped with a collar for some hours to avoid self sniffing.

Groups of six mice were sacrificed 0, 48, 57, 72, 120 and 168 hours following immunization. Iliac, cervical lymph nodes and spleen were individually harvested from each mouse. Single-cell suspensions from cervical, iliac lymph nodes and spleens were obtained as previously described [29].

In other experiments, purified anti-CD62L (MEL-14, Southern Biotechnology, USA) was administered intravenously at 100 µg/mouse 6 h after immunization with OVA plus CpG ODN1826 and then every 24 h until harvest. Fifty-seven hours after immunization, lymph nodes and spleens were collected and analysed by flow cytometry.

### Flow cytometric analysis

Cell suspensions from lymph nodes and spleens were incubated with Fc-blocking solution [0.5 mg CD16/CD32 mAb (clone 93) (eBioscience, USA), 5% mouse serum, 5% rat serum, 0.2% sodium azide (all from Sigma-Aldrich) in 100 ml of HBSS] for 30 min at 4°C. T cells were stained with PerCP-conjugated anti-mouse CD4 (clone RM 4-5) (BD Pharmingen), APC-conjugated anti-mouse CD44 (clone IM7), CD45RB (clone C363.16A) or CD62L (Ly-22) (clone MEL-14) (all from eBioscience) for 30 min at 4°C.

For DCs studies, cells were labelled with eFluor 710-conjugated I-A/I-E (clone M5/114.15.2) and anti-mouse CD11c PE-conjugated (clone N418) (all from eBioscience).

CountBright absolute counting beads (Invitrogen, Molecular Probes, Oregon, USA) were used for counting the number of lymphocytes in each sample. All samples were analyzed by flow cytometry (FACScalibur, Becton Dickinson, San Diego, CA). Data analysis was performed by using Flow Jo software (Tree Star, Ashland, OR, USA).

### Statistical analysis

The number of proliferating T cells in lymph nodes and spleen was analysed individually and values were expressed as mean ± standard error of the mean (SEM).

Statistical differences between the quantity of antigen-specific transgenic T cells detected in lymphoid organs at different time points were assessed using one-way analysis of variance (ANOVA) and Tukey's post test for multiple comparisons. Statistical significance was defined as *P*<0.05. Graphpad 4.0 software was used for analysis.

### Mathematical model and assumptions

A multi-type Galton Watson (MGW) branching process was used to model *in vivo* CFSE proliferation data. The key assumptions featuring MGW models are: 1) each cell action is independent from others; 2) each dividing cell generates its own branching process; 3) the cell has no memory about previous time-steps (Markovian process) [37], [44], [45].

During each time-step Δ*t*, it is assumed that a single cell in generation *i*, *i* = 0,…., *p* (where *p* is the highest number of cell generation tracked by flow cytometry), can divide with probability γ*_i_* into two *i*+1 generation cells, rest in its generation *i* with probability δ*_i_*, or die/migrate with probability α*_i_*. Of course, 2 out of the 3 probability parameters can vary independently, e.g. δ*_i_* and γ*_i_*, being α*_i_* = 1−δ*_i_*−γ*_i_*. We assume that the process has distinct probabilities for cells in generations 0, 1, 2 [36], while probabilities for generations *i*>2 are assumed equal to those of generation *i* = 2. The MGW process is then specified by *i)* the choice of the time-step Δ*t*, *ii)* the initial cell number in generation 0 and *iii)* the vector of parameters that determine the probabilities for each cell generation, i.e., u = [δ_0_, γ_0_, δ_1_, γ_1_, δ_2_, γ_2_]*^T^*. For this process, we can define the first and second order moments, i.e. the mean and covariance matrix of live cells counts at each time-point *t*, which in turn, can be exploited to compute the Maximum Likelihood function for the estimation problem of the parameter u [39].

However, *in vivo* experiments shown at least two factors which violate the classical hypothesis on MGW models and the related inferences from experimental data, requiring specific modeling interventions. We outline below these items and the actions taken to overcome them:

1. The draining site is not ‘isolated’ with respect to other lymph nodes. This means that the cell proliferation process may be seriously affected by migration from and to other distal lymph nodes. In this case, the process division, death and quiescence rates are biased, and it is not true anymore that their sum is 1.
2. All the *in vivo* experiments require animal sacrifice to collect CFSE data. This means that measurements taken at different time points actually refer to different individuals. This fact introduces an inter-individual stochastic aspect which is not considered in classical inference schemes.

With reference to the first issue, the impact of T-cell egress has been ruled out, because the measurements, used for inference, were carried out at early time points when most of primed T cells have not left the draining lymph node. On the contrary, it is well known that a significant entry phenomenon of naïve T cells takes place over the measurement time horizon, due to the recruitment of T cells from other lymphoid sites. This problem has been solved by using a 2 stage procedure. First, the pure MGW model (with isolated draining lymph node) is exploited to compute the probability parameters on an appropriate experiment, where access is blocked through the treatment with the anti-CD62L antibody (see section on Results). Second, the MGW model is generalized by adding an immigration term *r* [46] to the naïve T cell population in the draining site. The generalized model can be estimated by using the experimental data and exploiting the MGW model used in the first stage.

Regarding the intrinsic difficulties of the second issue, model identification has been performed by using relative frequencies as model variables instead of cell counts. Relative frequency was calculated as the percentage of OVA-specific CD4^+^ CFSE^+^ T cells present in each generation towards the total number of OVA-specific CD4^+^ T cells present in the lymph node. This choice allowed us to derive an explicit expression for the Maximum Likelihood function for the MGW model, since the distribution of relative frequencies at different time points is asymptotically normal, and the number of initial cells starting the proliferation process is large [39]. In order to derive estimates û and 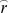 for the u and *r* MGW model parameters, we constructed the following iterative estimation procedure. We assume that an initial estimate of the MGW model u = û_0_ is available and proceed with the following steps: i) compute the mean value of T-cell counts of the MGW model for u = û and relative frequencies in each generation as a function of the parameter *r*; ii) estimate 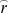 through a least squares criterion using the measurements at *t* = 48 and *t* = 57; iii) set *r =* 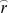 and compute the first and second order moments of the MGW with immigration terms; iv) update the estimate û by maximizing the new Maximum Likelihood Function of the relative frequencies data. The estimation procedure is then repeated until convergence of û and 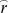.

## Results

### Antigen-loaded DCs are localized in draining lymph nodes

**Figure 1:**
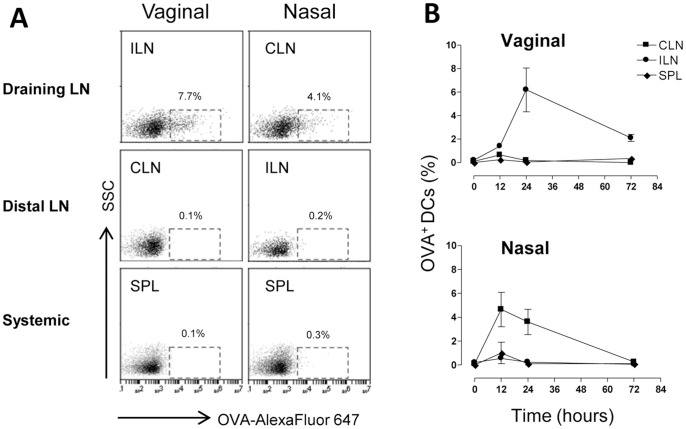
Antigen uptake by DCs following vaginal or nasal immunization. C57BL/6 mice were immunized by the vaginal or nasal route with OVA Alexa Fluor 647-conjugate (25 µg/mouse) and CpG ODN (20 µg/mouse). Groups of animals were sacrificed 0, 12, 24 and 72 hours after mucosal immunization and iliac lymph nodes (considered draining after vaginal and distal after nasal immunization respectively), cervical lymph nodes (distal after vaginal and draining after nasal immunization respectively) and spleen were collected. DCs positive for fluorescent OVA were analysed by flow cytometry. Shown is the percentage of OVA-Alexa Fluor 647 positive cells among total CD11c^+^ MHC class II^+^ cells.

The localization of antigen-loaded DCs was assessed after vaginal and nasal administration of the vaccine model formulation. To this aim mice were inoculated vaginally or nasally with OVA-Alexa Fluor 647 conjugate and the mucosal adjuvant CpG ODN 1826. Mice were pre-treated with progesterone in order to synchronize the estrous cycle to the diestrous phase and to facilitate antigen-uptake. Antigen loading was analyzed following vaginal or nasal immunization in draining lymph nodes (iliac and cervical lymph nodes respectively), in distal lymph nodes (cervical and iliac lymph nodes, respectively) and in the spleen. The presence of antigen-loaded DCs in lymphoid sites was assessed 0, 12, 24 and 72 hours after the inoculum. Within the first day after the immunization, antigen-loaded DCs (CD11c^+^ MHC class II^+^ cells) were localised only in the respective draining lymph nodes, with about 6% and 4% of DCs detected following vaginal and nasal immunization respectively (Fig. 1 A and B). On the contrary, in distal lymph nodes and in the spleen, antigen-loaded DCs were not detected at any time point (Fig. 1 A and B), indicating that also following vaginal immunization antigen presentation occurs within lymph nodes draining the immunization site, and not in other lymphoid sites, in accordance with what was observed and previously reported for nasal immunization [32]. No significant differences in the number of antigen-loaded DCs were indeed observed between vaginally and nasally immunized mice. These data show that the antigen-uptake following vaginal immunization of progesterone-treated mice was as efficient as the one observed following nasal immunization.

### Vaginal immunization elicits a rapid recruitment and clonal expansion of CD4^+^ T cells in draining lymph nodes

**Figure 2:**
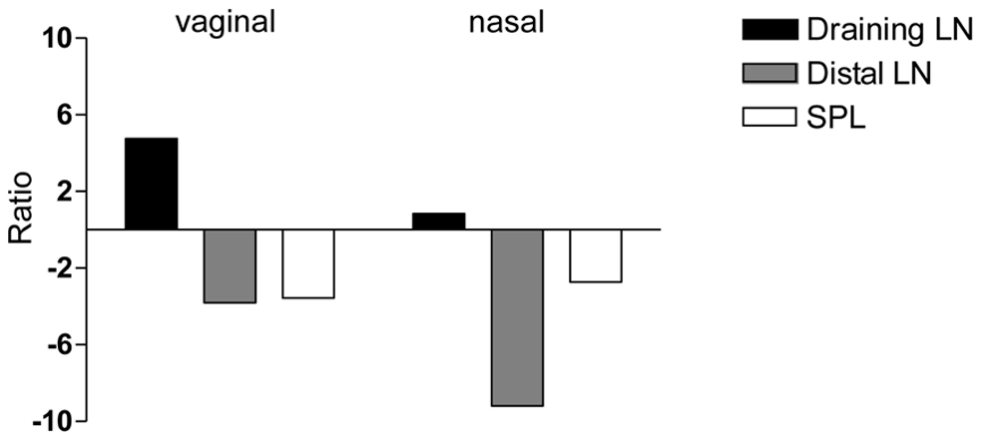
Antigen-specific T-cell distribution in draining, distal lymph nodes and spleen following vaginal or nasal immunization. CFSE-labelled OT-II CD4^+^ T cells were transferred into recipient C57BL/6J mice. Twenty-four hours later, recipient mice were immunized with OVA (25 µg/mouse) and CpG ODN 1826 (20 µg/mouse) by the vaginal or nasal route. The average number of OVA-specific CD4^+^ T cells was calculated in draining, distal lymph nodes and spleen of mice 57 hours following vaginal or nasal immunization or in naïve mice. Here the ratio between the absolute number of OVA-specific CD4^+^ T cells detected in lymphoid organs of mice immunized by the vaginal or nasal route *versus* naïve mice is reported.

To characterize T-cell clonal expansion and distribution, OVA-specific transgenic CD4^+^ T cells, labeled with CFSE, were adoptively transferred into recipient mice that were immunized with the model formulation OVA and CpG ODN 1826 by the vaginal or nasal route. The distribution of OVA-specific transgenic T cells in adoptively transferred recipient mice was assessed in draining and distal lymph nodes and spleen, 57 and 72 hours after immunization and compared with the one detected at the time the antigenic stimulus was given (time 0) [47] (Table 1). Following vaginal immunization was observed a 5-fold increase of antigen-specific CD4^+^ T cells in draining iliac lymph nodes at 57 hours (Fig. 2 and Table 1), and a 7-fold increase at 72 hours (1×10∧5 OVA-specific CD4^+^ T cells respect to 1.6×10∧4 at time 0; *P*<0.001; Table 1). The amount of total lymphocytes also increased gradually from time 0 to 72 hours, suggesting a general recruitment of lymphocytes from other lymphoid sites (Table 1). On the contrary, in distal cervical lymph nodes, a 4 fold decrease was detected at 57 hours (8.8×10∧3 respect to 3×10∧4 at time 0; *P*<0.05; Table 1 and Fig. 2). The distribution of the antigen-specific T cells described was comparable to the one observed following nasal immunisation, where the recruitment of antigen-specific T cells into draining sites appeared slower with a 3-fold increase observed at 72 hours (8×10∧4 respect to 3×10∧4 at time 0; *P*<0.05; Table 1) while, a drastic reduction of antigen-specific T-cell number was detected in distal iliac lymph nodes already 57 hours after immunization (1.7×10∧3 respect to 1.5×10∧4 at time 0; *P*<0.001; Table 1 and Fig. 2). In the spleen, a 3-fold decrease of antigen-specific T-cell was observed 57 hours following both vaginal and nasal immunization (Fig. 2), probably due to CD4^+^ T-cell recruitment in draining lymph nodes, while antigen-specific T-cell numbers were reestablished at 72 hours (Table 1). These data demonstrate a strong recruitment of antigen-specific T cells into the lymph nodes draining the immunization site, that was more rapid following vaginal than nasal immunization.

**Table 1:** Number of OVA-specific CD4^+^ T cells and total lymphocytes.

Route of immunization	Lymphoid organ^a^	Time	OVA-specific T cells^b^	Total Lymphocytes
Vaginal	ILN	0	15.7±6.2	2303±925
		57	74.5±34.5	4385±1300
		72	106±64*** c	5017±2600
	CLN	0	31.9±21	4264±1322
		57	8.8±3.6 *	3890±1190
		72	16±9.1	4888±2400
	SPL	0	185.5±24.5	40748±11482
		57	52.2±19 ***	30052±5117
		72	157±70	47902±35887
Nasal	CLN	0	31.9±21	4264±1322
		57	26±28	4832±1219
		72	79.5±47.6 *	7636±3777
	ILN	0	15.7±6.2	2303±925
		57	1.7±0.7 ***	1909±541
		72	4.5±3.2 ***	1735±1059
	SPL	0	185.5±24.5	40748±11482
		57	68.2±16.4 *	47167±10180
		72	186.4±118	43000±15916

a ILN: Iliac lymph nodes (subiliac, medial and external), CLN: cervical lymph nodes (superficial and posterior) and SPL: spleen.

b Absolute number of OVA-specific CD4^+^ T cells calculated as [(percentage of CD4^+^CFSE^+^×absolute number of lymphocytes)/100] for each lymphoid organ. OVA-specific T cells and total lymphocytes absolute numbers are to be multiplied×10∧3.

c One-way analysis of variance (ANOVA) and Tukey's post test were used to compare the number of OVA-specific CD4^+^ T cells present in lymphoid organs at different time points.

* *P*<0.05;

*** *P*<0.001.

**Figure 3:**
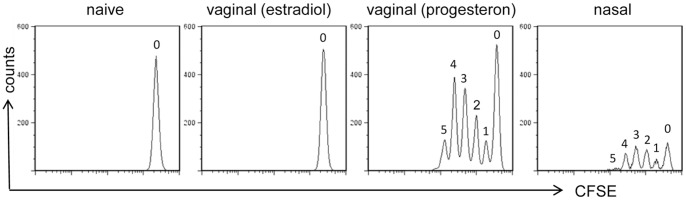
Clonal expansion of OVA-specific CD4^+^ T cells following vaginal or nasal immunization. OVA-specific proliferation of CD4^+^ T cells was assessed 57 hours following immunization in draining lymph nodes (iliac for vaginally and cervical for nasally immunized mice, respectively) and analyzed as CFSE dilution (x axis) on the gated CFSE^+^ CD4^+^ populations with light scatter properties of lymphocytes. The number of respective cell generation is reported for each peak. Results are representative of two experiments performed with six animals per group.

Fifty-seven hours following immunization of progesterone treated mice, antigen-specific T cells initiated their clonal expansion within draining lymph nodes, as identified by CFSE dilution (Fig. 3). Five cell generations were detectable in iliac lymph nodes of vaginally immunized mice and 4 major in cervical lymph nodes of nasally immunized mice (Fig. 3). The presence of an additional peak in vaginally immunized mice is consistent with the observed more rapid T-cell recruitment. On the contrary, no T-cell proliferation was observed in estrogen treated mice, since antigen-uptake does not occur in the vaginal tissue in the estrous phase, confirming the hormonal dependence of T-cell priming in the genital tract [19]. Untreated animals maintained a high level of CFSE fluorescence in lymph nodes, indicating that no cell division occurred (Fig. 3).

### CFSE proliferation data were analysed using mathematical model based on Multy-type Galton Watson branching process

**Figure 4:**
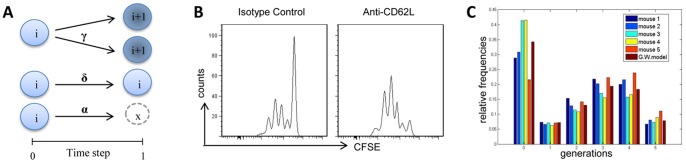
A Galton Watson branching process in the discrete time. A) Schematic representation of the stochastic behavior of the branching process. The *i* in the circle denotes the cell generation tracked with CFSE. Between two subsequent time-steps a single cell in *i* generation could take independently by the other cells, three biological decisions: entering in division with probability γ*_i_* and creating two cells of *i*+1 generation, resting undivided in generation *i* with probability δ*_i_*, dying with probability α*_i_* = 1−δ*_i_*−γ*_i_*. The formalism allows us to calculate the moments of cell counts. B) Proliferation of antigen-specific CD4^+^ T cells following treatment with isotype control or anti-CD62L antibody. Clonal division was assessed in draining lymph nodes 57 hours following immunization and analyzed as CFSE dilution (x axis) on the gated CFSE^+^ CD4^+^ populations with light scatter properties of lymphocytes. C) Comparison of the experimental relative frequencies obtained from 5 mice at the time-point *t* = 57 h and the predicted values by the branching process for each cell generation following vaginal immunization.

A mathematical model based on the MGW branching process with immigration was adopted to analyse antigen-specific CD4^+^ T-cell proliferation data in draining lymph nodes following vaginal and nasal immunization. During each time-step it is assumed that a single cell in generation *i*, can make three independent probabilistic choices: divide with probability γ*_i_* into two *i*+1 generation cells, rest in its generation *i* with probability δ*_i_* or die/migrate with probability α*_i_*. A schematic representation of this process is reported in Fig. 4A.

Since a lymph node is not an ‘isolated’ site but it is a component of the complex immunologic network including different lymphoid sites, T-cell proliferation process may be influenced by the immigration from and the migration to distal lymph nodes with a consequent bias on the division, death and quiescence rates. In our case the migration component was excluded since measurements were carried out at the time point (57 h) when most of primed T cells have not left the draining lymph nodes. The continuous recruitment of naïve T cells into draining lymph nodes from other lymphoid organs was considered by introducing an ‘immigration’ additive term in the MGW model, represented by a parameter *r*, that indicates the number of naïve T cells entering the draining lymph node during each time step (immigration parameter). We assumed as initial cell number, the amount of antigen-specific T cells detected in a lymphoid organ at the time when the antigenic stimulus was given [47]. The starting time point for the counting process (*n* = 0) was fixed at *t = 42* hours, the time that is considered as the average time when a cell begins to divide. The model time step was chosen as Δ*t = 3* hours based on the assessment that three new cell generations were observed in draining lymph nodes between 48 and 57 hours following vaginal and nasal immunization (data not shown). Different probability parameters were assumed for generations *i* = 0, 1 and 2 while parameters for generations >2 were assumed equal to those for *i* = 2 [36]. A Maximum Likelihood approach was adopted to estimate model parameters including both the probability and the immigration parameters. The parameters were estimated on the number of OVA-specific CD4^+^ T cells in each cell generation, reported as a relative frequency (percentage of CD4^+^ CFSE^+^ cells per absolute number of lymphocytes/100). Data from a total of 20 animals immunized by the vaginal or nasal route and sacrificed at time 0 or 57 were used for the model analysis. To improve the estimate quality, an additional experiment was performed blocking T-cell entry into lymph nodes and providing data useful for an initial evaluation of the probability parameters (û_0_ in Material and Methods Section). In this experiment, mice, previously adoptively transferred with OVA-specific CD4^+^ T cells, were immunized with OVA and CpG ODN and daily treated with anti-CD62L antibody. As shown in Figure 4B, the anti-CD62L antibody treatment prevented the entry of naïve antigen-specific T cells within the draining lymph nodes.

The estimated immigration parameter 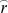 was 3280 following vaginal immunisation and 915 following the nasal route. The probability parameter vector u and the corresponding standard deviations obtained from analyzing T-cell proliferation following vaginal and nasal immunization are reported in Table 2. The parameters were estimated for undivided cells (δ_0_ and γ_0_), first generation (δ_1_ and γ*_1_*) and second and subsequent generations (δ_2_ and γ_2_). The values of parameters γ*_i_*, representing the probability of a cell to proliferate, calculated for i = 0, 1, 2 increased with increasing number of cell generations and were higher following vaginal than nasal immunisation. At the same time, the parameters δ*_i_*, representing the probability of a cell to rest in the same generation, were lower for vaginal than nasal immunisation. These data suggest that after vaginal immunisation antigen-specific T cells rapidly increase their ability to divide with increasing number of cell divisions. Moreover, the parameters α*_i_* for i = 0, 1, 2 (probability for a cell to die/migrate in each cell division), calculates as α*_i_* = 1−δ*_i_*−γ*_i_* decreased with increasing number of cell generations, suggesting a reduced probability to die/migrate.

**Table 2:** MGW process parameter estimates.

Route	Parameters	Values	Standard Deviation
Vaginal	δ_0_	0.38	0.03
	γ_0_	0.15	0.0483
	δ_1_	0.47	0.16
	γ_1_	0.26	0.124
	δ_2_	0.37	0.10
	γ_2_	0.50	0.126
Nasal	δ_0_	0.32	0.09
	γ_0_	0.11	0.0432
	δ_1_	0.40	0.13
	γ_1_	0.21	0.11
	δ_2_	0.55	0.18
	γ_2_	0.25	0.146

A snapshot of the experimental relative frequencies for each cell generation and the relative frequencies predicted by the identified model are reported in Figure 4C showing a satisfactory quality of fit represented by the low discrepancy between the model relative frequencies and data.

In conclusion, the modeling analysis shows that the developed model is reliable and can be fruitfully exploited for predictive simulations and/or other possible purposes related to the study of primary T-cell activation.

### Antigen-specific transgenic T cells express activation and homing molecules

**Figure 5:**
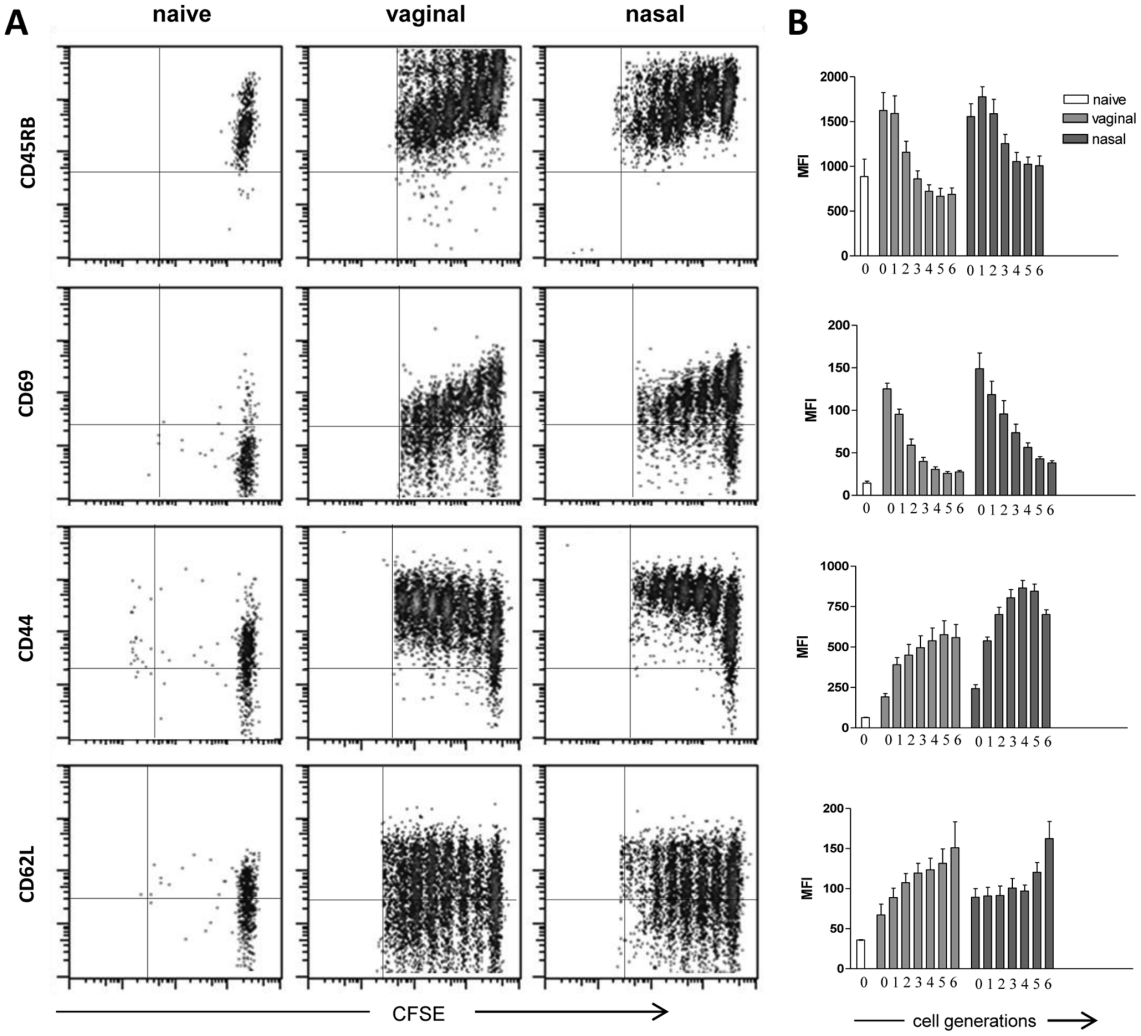
Phenotypic analysis of proliferating antigen-specific CD4^+^ T cells. The expression of CD45RB, CD69, CD44 and CD62L was analysed as a function of cell divisions on OVA-specific CD4^+^ T cells from draining lymph nodes 72 hours following vaginal or nasal immunization with OVA and CpG ODN 1826. A). Dot plot analysis of CFSE dilution (x axis) *versus* CD45RB, CD69, CD44 and CD62L expression (y axis) on OVA-specific CD4^+^ T cells in naïve and vaginally or nasally immunised mice. B). Mean fluorescence intensity of CD45RB, CD69, CD44 and CD62L expression per each cell generation of OVA-specific CD4^+^ T cells from naïve mice (open histogram) and vaginally or nasally immunised mice (dark and light gray respectively). Cell divisions (from 0 to 6) are identified by CFSE intensity dilutions. Phenotypic analysis was performed on single mice. Results are representative of two experiments with six animals per group.

The phenotype of transgenic CD4^+^ T cells was assessed in draining lymph nodes three days following vaginal or nasal immunization. The expression of surface markers, in immunized or naïve mice, was analysed in each generation (from 0 to 6) identified by CFSE fluorescence intensity (Fig. 5A) and the respective MFI values were reported in Fig. 5B. The modulation of surface marker expression in transgenic CD4^+^ T cells was very similar following vaginal and nasal immunization, with only small differences in MFI values (Fig. 5A–B). Undivided antigen-specific transgenic T cells (generation 0) still expressed high levels of the naïve CD45RB marker that was down-modulated in proliferating T cells (Fig. 5A–B). The early activation marker CD69 was rapidly up-regulated within the first cell cycle, with about a 10 fold increase of the MFI compared to naïve T cells, and then decreased with advancing cycles of cell division (Fig. 5A–B). The expression of the activation marker CD44 was increased gradually throughout the cell generations maintaining a high level of expression in the last generation observed (Fig. 5A–B). The expression of CD62L was low in CD4^+^ T cells from naïve mice, while it was up-regulated in immunized mice with a fourfold increase in the most advanced cell divisions (Fig. 5A–B).

The phenotypic analysis of antigen-specific proliferating CD4^+^ T cells confirmed that, following both vaginal and nasal immunization, T cells were activated by the antigen encounter and were able to acquire homing molecules to disseminate towards distal lymphoid organs, as previously described following intranasal immunization with a recombinant bacterial vector [29], [31].

### Antigen-specific CD4^+^ T cells disseminate to distal lymphoid organs

**Figure 6:**
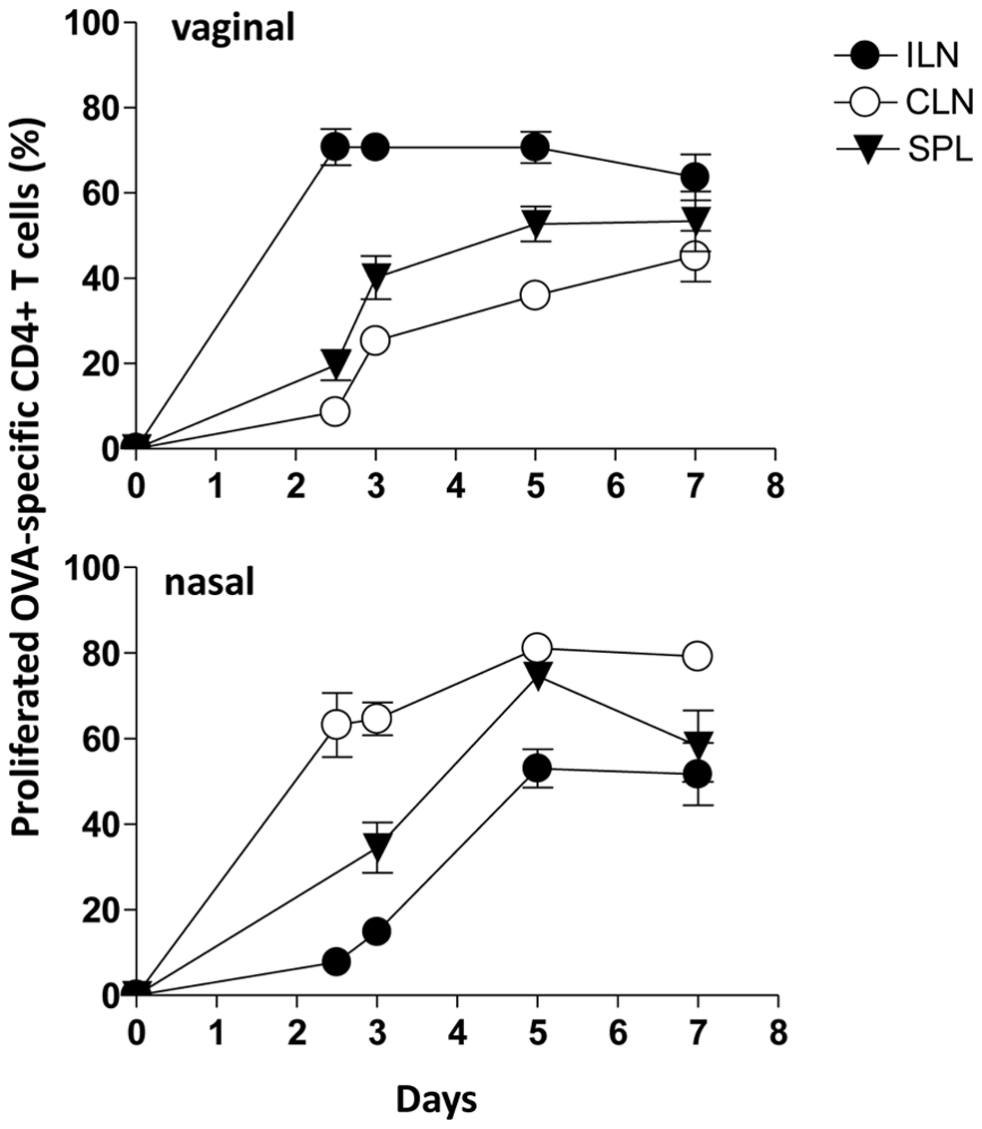
Time course analysis of the OVA-specific CD4^+^ T-cell clonal expansion following vaginal or nasal immunization. The clonal expansion of adoptively transferred CD4^+^ T cells following vaginal or nasal immunization with OVA plus CpG ODN 1826 was analyzed on days 2.5, 3, 5, and 7 post immunization in draining and distal lymph nodes and spleen. Values reported in y axis indicate the percentage of proliferating antigen-specific T cells. OVA-specific T cells were identified as CD4^+^ CFSE-positive populations, with light scatter properties of lymphocytes. The percentages of proliferating cells are shown on the *y* axis. Bars represent the means ± standard errors of the means (SEM) of values from nine mice assessed in three separate experiments.

OVA-specific CD4^+^ T-cell proliferation was analysed at different time points (0, 2.5, 3, 5, and 7 days) following vaginal or nasal immunization in the respective draining and distal lymph nodes and spleen. The kinetic analysis of the T-cell priming showed a rapid antigen-specific clonal division of transgenic CD4^+^ T cells in the respective draining lymph nodes already at day 2.5, with about 75% of proliferating transgenic T cells in iliac and 60% in cervical lymph nodes following vaginal or nasal immunization respectively (Fig. 6). Proliferation values were almost steady maintained at all analysed time points. The dissemination of proliferated T cells into distal lymph nodes started at day 3 and increased at day 5 with about 35% of vaginally primed T cells in distal cervical lymph nodes and 50% of nasally primed T cells in distal iliac lymph nodes (Fig. 6). The percentage of proliferated cells in the spleen was about 50% and 75% of transgenic T cells following vaginal and nasal immunization respectively (Fig. 6). Seven days after immunization, divided T cells were still detectable in distal lymph nodes and in the spleen. No significant differences in the percentage of T-cell proliferation were observed following vaginal and nasal immunization at any time-points analysed in draining and distal lymph nodes and spleen.

## Discussion

In the present work antigen-specific CD4^+^ T-cell primary activation and dissemination following vaginal immunization was characterized and compared to the one elicited by the nasal route. The very early events of CD4^+^ T cell clonal expansion following immunization by the two mucosal routes were analysed applying a Multi-type Galton Watson branching process. Understanding in depth the early events that lead to the immune response is crucial for the rational development of appropriate prime-boost vaccine strategies.

Following vaginal immunisation of mice treated with progesterone, antigen-bearing DCs were detected only in draining lymph nodes and not in distal lymph nodes or in the spleen, in line with what observed with nasal immunization [32]. In vaginally immunized mice, antigen-loaded DCs persisted in draining lymph nodes up to 72 hours while following nasal immunisation were detected only within 24 hours. The extended persistence following vaginal immunisation is probably due to the fact that mice were immunized in the diestrous phase, when the antigen uptake is enhanced [17], [19], [48]–[50].

A recruitment of antigen-specific T cells from distal lymphoid sites was observed in draining lymph nodes with a simultaneous significant decrease of T cells in distal lymph nodes and in the spleen. Vaginal immunization of mice was more effective at stimulating antigen-specific CD4^+^ T-cell recall in draining lymph nodes compared to nasal immunization. The analysis of T-cell clonal expansion showed 5 cell generations, while at the same time-point 4 cell generations were detected in cervical lymph nodes following nasal immunization. These results are in line with what reported by Marks and colleagues showing higher frequency of antigen-specific T cells in draining iliac lymph nodes following vaginal respect to nasal immunization with OVA and cholera toxin [19]. This more rapid local T-cell proliferation following vaginal immunization can be due to the lack of an organized lymphoid tissue in the female genital tract. Indeed, upon vaginal immunization, iliac lymph nodes directly drain the genital mucosa [5], whereas following nasal vaccination antigen-specific T-cell proliferation first takes place in the NALT and then in cervical lymph nodes, as previously showed by our group and others [30], [51]. The T-cell proliferation observed following vaginal immunization was strictly dependent on the estrous phase since no T-cell response was observed in estrogen treated mice. This is in accordance with previous studies reporting that immune response in the female genital tract depends upon the reproductive hormones [3], [18], [22], [52].

To characterize in depth the antigen-specific T-cell clonal expansion in draining lymph nodes in the first hours following antigen administration, a mathematical model, based on the MGW branching process with immigration was exploited as a tool to estimate *in vivo* the probability of an antigen-specific CD4^+^ T cell to divide following the antigen-encounter. While this model has been previously applied for *in vitro* studies [38]–[40], it had not been previously applied to *in vivo* proliferation data. This is due to the fact that *in vivo* analysis raises several difficulties, mainly due to the fact that i) a lymph node is not an ‘isolated’ site but is part of a complex system and ii) data are collected from different animals introducing a inter-individual stochastic aspect. The impact of T-cell egress has been ruled out by carrying out the measurements, used for inference, at early time points (57 hours) when almost no T-cell has left the draining lymph node. Nevertheless, a significant recruitment of naïve T cells from other lymphoid sites takes place over the measurement time horizon. To address this problem, the MGW model was generalized by adding an immigration term [46] calculated with an appropriate experiment where access of naive T cells was blocked through the treatment with the anti-CD62L antibody. Concerning the second point, model identification has been performed by using relative frequencies as variables instead of cell counts. This choice allowed us to derive an explicit expression for the Maximum Likelihood function for the MGW model since the distribution of relative frequencies at different time points is asymptotically normal, provided the number of initial cells starting the proliferation process is large [39]. The good quality of fit, represented by the low discrepancy between the model relative frequencies and experimental data, shows that the developed model is reliable and can be therefore fruitfully exploited for predictive simulations and/or other possible purposes related to the study of primary T-cell activation. Model parameter estimates indicate that the probability of a cell to proliferate was higher following vaginal than nasal immunization, suggesting that the vaginal immunization route induces a higher CD4^+^ T-cell proliferation in draining lymph nodes with respect to the nasal route. Ongoing work is focused on modeling the whole complex immunological network, including both draining, distal lymph nodes and spleen, in order to get further quantitative information and create a model capable of predicting the intensity and distribution of T cells in the various lymphoid organs.

Proliferated antigen-specific CD4^+^ T cells were activated by the antigen encounter, as shown by the rapid expression of the early activation marker CD69 [53] and CD44 [54] within the very early cell generations. On the contrary, CD45RB, a marker expressed by naïve T cells [55], was down-modulated as cell division proceeded. The expression of CD62L, a molecule essential for lymphocyte entry into lymph nodes [56], was gradually increased by proliferated T cells. The phenotypic analysis of antigen-specific proliferating CD4^+^ T cells in draining lymph nodes showed a similar modulation pattern of surface marker following vaginal and nasal immunization confirming that T cells were activated and able to disseminate into distal lymph nodes. These findings are in line with precedent data obtained following intranasal immunization with recombinant bacteria [29], [31]. Despite T-cell recruitment and antigen-specific T-cell proliferation that was faster in draining lymph nodes following vaginal immunisation, no statistically significant differences in the percentage of antigen-specific proliferated CD4^+^ T cells were observed at later time-points (5 and 7 days) between vaginal and nasal immunization.

All together these data show that not only nasal but also vaginal immunization, despite the absence of an organized mucosal associated inductive site in the genital tract, is very efficient in priming antigen-specific CD4^+^ T cells in draining lymph nodes and inducing their dissemination from draining lymph nodes towards distal lymphoid organs.
